# 铜死亡在血液系统肿瘤中的研究进展

**DOI:** 10.3760/cma.j.cn121090-20240327-00116

**Published:** 2024-10

**Authors:** 灵杰 周, 艳英 李, 柳芸 张, 娟 张

**Affiliations:** 1 电子科技大学医学院，成都 610054 School of Medicine, University of Electronic Science and Technology of China, Chengdu 610054, China; 2 四川省医学科学院·四川省人民医院（电子科技大学附属医院），临床医学检验中心及人类疾病基因研究四川省重点实验室，成都 610072 Department of Laboratory Medicine and Sichuan Provincial Key Laboratory for Human Disease Gene Study, Sichuan Provincial People's Hospital, School of Medicine, University of Electronic Science and Technology of China, Chengdu 610072, China

## Abstract

铜死亡是一组不同于凋亡、坏死性凋亡和铁死亡的独立死亡形式，由铜所介导，主要影响线粒体三羧酸（TCA）循环中蛋白酶硫辛酰化并通过寡聚表现出细胞毒性，但其具体机制、信号转导过程和调控的方式仍然不清楚。线粒体在铜死亡发生发展中处于中心地位，影响细胞对铜毒性的敏感性。近年来，血液系统肿瘤通过靶向治疗与免疫治疗取得了较好的缓解，但仍存在复发率较高及预后较差的问题，因此亟需找到更加完善的预后指标以及开辟新的治疗思路。本文总结了铜与线粒体在肿瘤发生发展中的交互作用，为进一步探究铜死亡的机制以及应对血液系统肿瘤提供思路。

## Abstract (en)

Cuproptosis is a type of independent cell death form, that differs from apoptosis, necroptosis and ferroptosis. It is mediated by Copper（Cu）, and mainly affects the lipoylation of proteases in the mitochondrial tricarboxylic acid（TCA）cycle and exhibits cytotoxicity through oligomerization; however, its specific mechanism, signal transduction process and regulation mode are still not clear. Mitochondria affect the sensitivity of cells to copper toxicity and play a central role in the occurrence and development of copper-related death. In recent years, though hematological tumors have achieved better remission through targeted therapy and immunotherapy, they are associated with high recurrence rates and poor prognoses. It is therefore imperative to find better prognostic indicators and new treatment ideas. This paper summarizes the interaction between Cu and mitochondria in the development of tumors and provides ideas for further exploration of the mechanism of copper death and coping with hematological tumors.

一种新型的受调节的细胞死亡形式——铜死亡，具有铜依赖性，可能与各种癌症的发生过程有关。铜是人体内的微量元素，与多种信号通路和肿瘤相关的生物学行为密切相关。而过量的铜可导致细胞死亡，但铜诱导细胞死亡的机制和具体形式仍不清楚。最近的一项研究[1]表明，铜死亡是一种独立的细胞死亡形式，与线粒体呼吸和硫辛酸途径高度相关。越来越多的研究通过生物信息学分析来关注铜死亡与癌症之间的重要联系。Tsvetkov等[1]先前研究中鉴定并验证了与铜死亡相关的基因（CRG），大部分研究侧重于铜死亡关键基因（CKG）的表达水平与肿瘤预后、肿瘤发生发展以及铜死亡敏感性的关系。本文就铜死亡机制及铜与线粒体在肿瘤发生发展中的交互作用作一综述。

一、铜死亡与血液系统肿瘤的关系

铜是人体日常代谢所必需的微量元素，细胞外的铜由铜转运体1（CTR1）或溶质载体家族31成员1（SLC31A1）转运到细胞内，主要由铜蓝蛋白（CP）运输到肝脏，再与金属硫蛋白（MT）结合后储存，多余的铜通过ATP酶铜转运体（ATP7A/B）排出体外[2]。线粒体是铜的主要储存和利用部位，大部分铜参与铜酶的合成，协助产生ATP，维持氧化还原稳态。

血液系统肿瘤是一组与血液、骨髓和器官相关的疾病，包括多发性骨髓瘤（MM）、淋巴瘤和白血病等在内的造血系统疾病，具有恶性程度高、治疗复杂和预后差等特点[3]。与健康个体相比，血液系统肿瘤患者血清铜水平升高，且血清铜浓度与其预后密切相关[4]。铜可促进肿瘤的进展、转移、血管形成和代谢调节[2]。一方面，铜直接激活血管内皮生长因子、成纤维细胞生长因子2、肿瘤坏死因子和IL-1等血管生成因子[5]，促进肿瘤血管的形成，还通过ATOX-ATP7A-LOX通路促进肿瘤转移[5]。另一方面，铜与许多激酶受体，包括PI3K/Akt、PDK-1、MEK1和MAPK等相互作用，促进肿瘤进展[6]；激活ULK1、ULK2[6]和改变c-Myc稳定性，从而影响自噬[2]；上调PD-L1表达，促使肿瘤细胞免疫逃逸[6]。但过多的铜在细胞内蓄积即铜超载，将导致细胞死亡，这种新的死亡形式被命名为铜死亡（图1A）。因此，铜具有双面性，一定浓度下促进肿瘤进展、转移，而过量积累又将导致铜死亡发生。

**图1:**
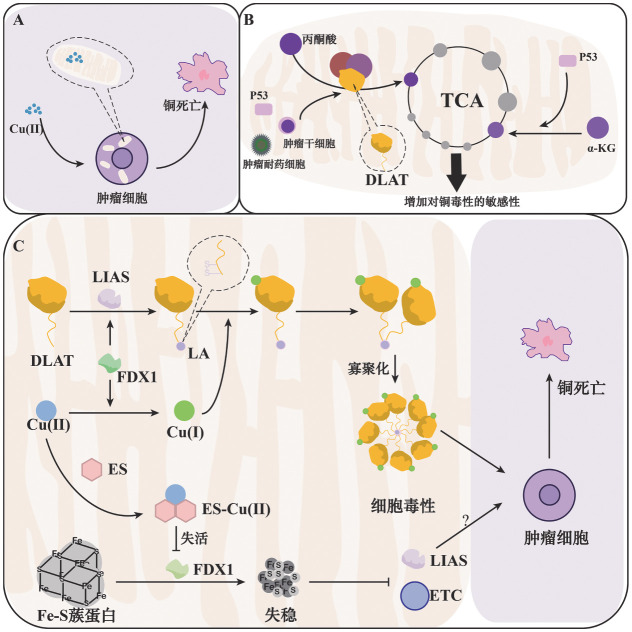
铜死亡相关机制通路图 **A** 过量铜进入细胞线粒体内造成铜超载，引发铜死亡；**B** p53、肿瘤耐药细胞和肿瘤干细胞提高碳源进入TCA循环，增加铜毒性敏感性，更易发生铜死亡；**C** 由FDX1还原的Cu^+^使得DLAT硫辛酰化并与Cu^+^结合，进而寡聚，产生细胞毒性，铜与ES结合后导致FDX1失活，不能维持Fe-S蔟蛋白稳定性导致铜死亡 **注** Cu：铜；DLAT：二氢脂酰胺S-乙酰基转移酶；LIAS：硫辛酸合成酶；FDX1：铁氧还蛋白1；LA：硫辛酰化；ES-Cu：一种铜与铜离子载体的复合体；ETC：电子传递链

二、铜死亡及其调控机制

最新研究表明铜死亡不同于目前已知的死亡形式，细胞凋亡、铁死亡、坏死性凋亡、氧化应激等死亡抑制剂均不能抑制铜死亡发生[1]。一方面，铜死亡与线粒体TCA循环酶密切相关，铜与二氢脂酰胺S-乙酰基转移酶（DLAT）结合使其发生硫辛酰化，又通过二硫键异常寡聚，进而产生细胞毒性导致细胞死亡[1]。其中，DLAT是丙酮酸脱氢酶复合体（PDH）的重要组成亚基，硫辛酰化的PDH复合体是调节碳源进入TCA循环的入口之一[7]，且已证实线粒体呼吸越活跃的细胞对铜毒性越敏感，更容易发生铜死亡（图1B）[1],[7]，因此铜死亡还被认为是一种代谢性细胞死亡。另一方面，铜通过降低Fe-S簇蛋白的稳定性，产生细胞毒性从而诱导铜死亡，但其具体机制尚不清楚，一些学者猜测可能与电子传递链和硫辛酸合成酶（LIAS）合成有关[1],[8]，具体如图1C所示。

在上述铜介导细胞死亡的过程当中，铁氧还蛋白1（FDX1）扮演了十分重要的角色，首先FDX1将Cu^2+^转化为毒性更强的Cu^+^，其次FDX1是硫辛酰化蛋白上游的调控因子，直接与LIAS结合，共同促进DLAT发生硫辛酰化[1]。对于Fe-S簇蛋白而言，FDX1作为还原剂促进了Fe-S簇蛋白的合成，而铜与铜离子载体的复合体［如伊利司莫（ES）-Cu］可直接作用于FDX1，抑制这个过程导致蛋白失稳[1]。但另一项研究认为，FDX1并未直接参与Fe-S簇蛋白合成，铁氧还蛋白2（FDX2）才是线粒体Fe-S簇蛋白合成中唯一的电子供体，FDX1只参与到LIAS依赖的硫辛酰化过程中[9]。蛋白硫辛酰化需要FDX介导，而FDX又作为还原剂维持Fe-S簇蛋白稳定，对于铜死亡机制而言，FDX的作用也具有双面性。

FDX1也可促进铜死亡发生。首先，FDX1表达水平反映了硫辛酰化蛋白的含量，含量越高越容易发生铜死亡；其次，FDX1缺失会导致ES介导的铜死亡产生抗性，这与FDX1促进铜从ES-Cu释放有关[10]。同样Tsvetkov等[1]认为这与FDX1作为ES的靶点，促进ES介导的细胞死亡相关。FDX1抑制细胞内氧化还原调节系统，降低烟酰胺腺嘌呤二核苷酸磷酸（NADPH）和谷胱甘肽（GSH）的水平，而GSH作为体内铜螯合剂，当其表达减少后游离铜增加，从而促进铜死亡[10]。此外，FDX1与糖代谢密切相关，通过基因集富集分析（GSEA）显示FDX1的表达与氧化磷酸化代谢通路呈正相关[11]，提示FDX1表达越高，线粒体呼吸越活跃，对铜毒性越敏感。究其原因，缺少FDX1会降低COX2与COX6A亚基水平，导致线粒体呼吸复合物COX Ⅳ活性降低，从而使线粒体呼吸转向糖酵解[9]。

除了FDX1外，P53通过调控相关基因的表达与酶的活性，以抑制糖酵解，促进脂肪酸氧化和谷氨酰胺转化为谷氨酸，进而促进糖酵解转为氧化磷酸化，同时，高水平的铜会激活癌细胞P53的表达，促进细胞凋亡，但细胞凋亡方式是否为铜死亡尚无定论[12]。

三、铜死亡与血液系统肿瘤的预后

铜死亡与血液系统肿瘤预后密切相关，可利用CRG构建风险模型以评估血液系统肿瘤的疗效和预后。CRG指与铜死亡发生相关的基因集，通常与能量代谢和金属稳态密切相关[13]。在铜死亡通路中，通过敲除全基因组，确定了与铜死亡功能相关的10个基因，这些基因的缺失可能导致铜死亡风险改变，因此它们被认为是CKG[1]。其中FDX1、LIAS、LIPT1、DLD、DLAT、PDHA1和PDHB促进铜死亡发生，而CDKN2A、MTF1和GLS抑制铜死亡发生[13]。

根据CRG建立的铜死亡相关预后风险评分（CRR）可作为独立预后因素，预测临床特征、免疫细胞浸润水平和患者预后。MM相关的研究[14]认为CRR预测国际分期系统（ISS）Ⅲ期患者的生存期更加准确，将CRR与MM常用的分期系统相结合，其可提高现有评估系统的预后预测能力。

在影响预后方面，CRR评分高的患者预后差，肿瘤微环境中免疫细胞浸润丰度低、发生免疫逃逸的可能性高、免疫治疗反应较差[14]–[19]。在急性髓系白血病（AML）中，铜死亡相关lncRNA与患者的总生存（OS）期密切相关，在预后与免疫应答方面与CRG一致[17]。此外，通过lncRNA构建lncRNA-miRNA-mRNA-ceRNA网络系统，相关lncRNA可调控miRNA，进而调节CRG的表达，影响患者预后[20]。

在调控免疫方面，CRG的RNA在巨噬细胞中富集，FDX1包含其中。miR-21-5p可以结合到FDX1的3′-UTR，抑制FDX1表达，进而调控肿瘤微环境中免疫细胞浸润[21]。在MM中，CRG评分与免疫检查点及肿瘤炎症指数（TIS）基因特征的表达水平呈负相关，TIS反映持续的适应性辅助性T细胞1（Th1）和细胞毒性CD8^+^ T细胞反应，高TIS意味着对抗PD-1/PD-L1药物的高反应性[15]。在AML中，MTF1、LIAS、ATP7B表达与相关免疫细胞呈正相关[20]。AML根据CRG表达不同分为3个表型，其中C3表型中CRG表达均明显下调，高度富集于免疫激活通路，但免疫细胞的抗肿瘤效率低，患者的生存率低[19]。在弥漫大B细胞淋巴瘤（DLBCL）中，当促进铜死亡的CRG高表达时，肿瘤微环境中免疫细胞浸润水平更高，预后也更好[16]。在MM、DLBCL和AML这三类血液系统肿瘤中，CDKN2A突变频率最高，CDKN2A能抑制免疫浸润、降低MM细胞FDX1的表达，同时与MM患者的耐药相关[15]–[18]。

在决定铜死亡敏感性方面，TCA循环主要富集在MM高风险组[15]。在DLBCL中，根据CRG的特点分为A、B亚型，其中B亚型主要富集p53信号通路和代谢相关通路[16]–[17]。还可根据共识簇将DLBCL分为3个亚型，其中OxPhos簇（OxPhos-DLBCL）显著富含线粒体OxPhos相关基因，较其他亚型有更多的碳源进入TCA循环，使线粒体供能增加[16]。在AML中，SF3B1突变型相比野生型对ES的敏感性更高，实体肿瘤的SF3B1突变与OxPhos减少相关，AML突变型对ES更敏感是否与线粒体呼吸相关有待验证[22]。对于其他线粒体呼吸活跃的血液系统肿瘤，如MM高风险组亚型、OxPhos-DLBCL和B亚型DLBCL[16]，是否也对铜毒性更加敏感，有待进一步验证。

综上，CRG在构建预后模型、评估患者生存预后、影响肿瘤发生发展以及决定铜死亡敏感性方面起着十分重要的作用，待其明确具体信号通路与调控机制后有望成为新的治疗靶标。

四、铜死亡在血液系统肿瘤的应用

根据氧化磷酸化越活跃越容易发生铜死亡的特点[1]，联合使用铜离子载体抗肿瘤具有远大前景。目前研究较多的两种铜离子载体：ES和双硫仑（DSF），扮演着将铜转运到细胞和线粒体内的角色，以此增加硫辛酰化的DLAT寡聚、导致Fe-S簇蛋白失稳，进而诱导细胞死亡。

1. ES：ES是一种铜离子载体，可降解ATP7A从而选择性将铜聚集在线粒体内，提高运载铜的效率[23]，肿瘤细胞对ES的敏感性与线粒体代谢活跃程度呈正相关。因此，ES对铂类、蛋白酶体抑制剂、分子靶向等耐药的肿瘤细胞和线粒体呼吸活跃的肿瘤细胞有着明显的杀伤力[8]。LDH活性升高与肿瘤糖酵解供能相关，因而血清LDH水平可作为评估患者对ES反应的潜在生物标志物。一项基于ES作用于AML的Ⅰ期临床试验[24]评估了其在复发或难治性AML患者中的安全性和有效性，并将剂量限制性不良反应定义为3级或4级非血液学不良反应。有研究认为ES与Cu^2+^结合进入肿瘤细胞后，使Cu^2+^还原为Cu^+^，这种氧化还原反应破坏线粒体呼吸，从而提高活性氧（ROS）水平，进而扰乱细胞能量的产生和代谢，以诱发肿瘤细胞的线粒体凋亡途径，导致细胞死亡[24]。但在治疗期间并未观察到羟基自由基增加和细胞线粒体膜电位变化，因此铜死亡可能是ES抗癌机制的一种合理解释。对于其他血液肿瘤：达雷妥尤单抗耐药的MM细胞，CD38表达下降，细胞内NAD^+^升高加强了线粒体代谢[25]；在维奈克拉耐药的淋巴瘤细胞中，凋亡调控因子（PUMA）表达的下调会引起线粒体代谢增加[26]，但ES对两种耐药肿瘤细胞的有效性还缺乏相关研究。此外，有体外实验结果[27]提示ES可联合糖酵解抑制剂（4-辛酯衣康酸）通过作用于NADPH来抑制糖酵解，进而促进铜死亡。

2. DSF：DSF是一种乙醛脱氢酶（ALDH）抑制剂，DSF-Cu复合体在血液系统肿瘤治疗中的有效性得到了证实。首先，DSF-Cu的代谢产物CUET通过聚集NPL4和诱导UPR显示出蛋白酶体样抑制作用[28]，蛋白酶体抑制剂是抗MM的主要药物之一，DSF-Cu在体内外都表现出强大的抗MM活性[29]，同时CUET可对抗铂类化学治疗药物的耐药性[30]。其次，DSF-Cu可通过抑制ALDH活性和降低转录蛋白水平来抑制干细胞样ALDH^+^ MM细胞生长[31]。此外，DSF-Cu可激活应激相关的ROS-JNK通路、抑制与生存相关的NRF2和NF-κB通路、使细胞周期停滞，从而抑制肿瘤细胞的增殖、集落的形成以及诱导细胞的凋亡[29],[32]。在抑制AML生长中，DSF也表现出了较好的效果，DSF-Cu通过抑制SOD1减少ROS的降解，从而促进细胞凋亡[33]；染色体NUP98-PHF23易位与AML患者低生存率有关，而DSF可促进NUP98-PHF23融合基因的AML细胞死亡，究其原因，DSF能够与PHF23的同源域PHD结合并与Cys残基反应，破坏NUP98融合结构，从而破坏组蛋白结合活性[34]。DSF-Cu通过下调MLL蛋白抑制急性淋巴细胞白血病肿瘤细胞集落形成和细胞生长[35]。在DLBCL中，DSF-Cu作用于AIP-BCL6-p53信号通路，降低BCL6的水平诱导DLBCL细胞凋亡[32]。DSF-Cu还通过诱导线粒体损伤，增加Bip蛋白的表达，抑制Akt通路激活内质网应激途径诱导骨髓增生异常综合征细胞死亡[36]。DSF-Cu可选择性地杀伤耐药和复发的肿瘤细胞群，为复发和耐药的患者提供一种新的治疗选择[28]–[30]。

五、总结与展望

血液系统肿瘤患者血清铜含量增高，参与一系列的信号通路促进肿瘤的生长和转移，加剧基因组的不稳定性，当过多的铜进入到细胞后又介导铜死亡发生，但尚没有相关研究明确两者的浓度界限。为了减少对正常细胞的损伤，将ES和铜封存在ROS的敏感聚合物中，只有进入肿瘤细胞后才释放铜，提高了作用效率[37]。此外，铜诱导细胞死亡信号通路的具体过程、调控方式以及与其他程序性死亡的关系，还有待进一步研究。在联合使用铁死亡诱导剂和铜离子载体后促进了铜死亡，这可能与铁死亡诱导剂抑制FDX1降解、GSH合成，提高蛋白质的硫辛酰化和寡聚有关[38]。CRG在不同肿瘤中发挥的作用不尽相同，具体的作用机制、调控方式以及与免疫浸润的关系均有待进一步研究，相关基因有望作为判断预后的因素和治疗的靶标。使用铜离子载体辅助治疗，理论分析可行性较高，但具体用到哪些细胞亚型，如何使用仍缺乏动物实验与临床试验研究佐证。总之，线粒体是铜死亡发生的中心，FDX1是关键调控因子，铜死亡联合化疗、免疫治疗作为辅助治疗，为抗血液系统肿瘤提供新的治疗思路。
